# Metal artifact reduction through MVCBCT and kVCT in radiotherapy

**DOI:** 10.1038/srep37608

**Published:** 2016-11-21

**Authors:** Gao Liugang, Sun Hongfei, Ni Xinye, Fang Mingming, Cao Zheng, Lin Tao

**Affiliations:** 1 Second People’s Hospital of Changzhou, Nanjing Medical University, Changzhou 213003, China; 2 Changzhou Cancer Hospital of Soochow University, Changzhou 213001, China; 3 The Third Affiliated Hospital of Anhui Medical University, Anhui 230000, China

## Abstract

This study proposes a new method for removal of metal artifacts from megavoltage cone beam computed tomography (MVCBCT) and kilovoltage CT (kVCT) images. Both images were combined to obtain prior image, which was forward projected to obtain surrogate data and replace metal trace in the uncorrected kVCT image. The corrected image was then reconstructed through filtered back projection. A similar radiotherapy plan was designed using the theoretical CT image, the uncorrected kVCT image, and the corrected image. The corrected images removed most metal artifacts, and the CT values were accurate. The corrected image also distinguished the hollow circular hole at the center of the metal. The uncorrected kVCT image did not display the internal structure of the metal, and the hole was misclassified as metal portion. Dose distribution calculated based on the corrected image was similar to that based on the theoretical CT image. The calculated dose distribution also evidently differed between the uncorrected kVCT image and the theoretical CT image. The use of the combined kVCT and MVCBCT to obtain the prior image can distinctly improve the quality of CT images containing large metal implants.

CT images are widely used in disease diagnosis and radiotherapy to locate the target area and normal tissues of patients and calculate absorbed dose for designing radiotherapy plans. The CT images of patients with metal implants contain severe metal artifacts. These artifacts are displayed as bright or dark areas and stripy patterns and can affect the accuracy of diagnosis and dose calculation in radiotherapy^1^,^2^,^3^. Hence, metal artifacts should be considerably reduced.

Several methods have been proposed for removal of metal artifacts from kVCT images^4^,^5^,^6^. These methods are categorized into modeling-based iterative reconstruction^7^,^8^,^9^ and sinogram correction^10^,^11^,^12^,^13^. In the former, a model is established by considering radiation, energy spectrum hardening, detector reception, and system noise and CT images are obtained through iterative reconstruction. This process is complicated and requires detailed understanding of the entire CT operation. Iterative reconstruction is also time-consuming and difficult to perform in clinical settings. In sinogram correction, projection through metals generates corrupted data, called metal trace. Generally, interpolation in the sinogram space or forward projection should be performed to estimate surrogate data. Interpolation-based methods^14^ are used to correct the corrupted projection data by using the uncorrupted data on the two sides of the metal trace. In interpolation, the surrogate data and the surrounding original uncorrupted data are not continuous and structure information in metal trace is lost; this method leads to introduction of new artifacts and loss of normal tissue structures when reducing metal artifacts. To overcome this limitation, Meyer^11^ proposed normalized metal artifact reduction (NMAR) method, in which a prior image is used to normalize the sinogram before interpolation. A prior image is an image without artifacts that can be obtained through segmentation and filtering of original CT images or preprocessing images. In forward projection of prior images, metal trace in the original CT image is replaced by surrogate data derived from forward projection of the prior image. The corrected sinogram is subjected to filtered back projection (FBP) to obtain the corrected image. However, inaccurate segmentation may affect the quality and accuracy of the prior image and is thus crucial in modification.

Different methods are used to eliminate metal artifacts from kVCT images for accurate diagnosis. In cases with large metal implants, the energy of kVCT rays is low, the detector receives low ray input, and the photon starvation effect is severe, resulting in severe artifacts in the CT image. Conversely, the energy of MVCBCT rays is high, and the rays can pass through the metal and be absorbed by the detector. In this technique, the effects of photon starvation and beam hardening are minimal and metal artifacts in the MVCBCT images are smaller than those in the kVCT images. However, the MVCBCT image contains higher noise and lower tissue resolution than those in the kVCT image. Hence, MVCBCT and kVCT images can be combined to reduce metal artifacts and improve image quality^15^,^16^,^17^. Paudel^18^ used MVCT images as prior images in the normalized reduction of metal artifacts from the kVCT image to correct artifacts and determine absorbed dose distribution. Jeon^19^ used MV sinogram to replace the metal-affected signals of the kV sonogram and employed FBP on the hybrid sinogram to obtain the corrected image. Wu^20^ completed kVCT projection data by using selectively acquired MVCBCT data; the image was reconstructed using FBP and iteration, and several metal artifacts caused by dental fillings and hip implants were removed from the CT images.

This paper proposes a new method for removal of artifacts. The metal portion in the MVCBCT image was segmented, and the metal-only image was forward projected to obtain metal trace. Prior image was obtained through fusion of MVCBCT and kVCT images and forward projection to acquire surrogate data. The metal trace in the kVCT image was replaced with the surrogate data to obtain the corrected sinogram. The corrected image was reconstructed through FBP using the corrected sinogram. Serious segmentation errors were avoided because the image was not segmented when obtaining the prior image. A similar radiotherapy plan was designed in Varian’s treatment plan system (TPS) by using the theoretical CT image, the uncorrected kVCT image, and the corrected image. Absorbed dose distribution was determined and compared between the uncorrected and corrected kVCT images. The effect of metal artifacts on radiotherapy was also analyzed.

## Materials and Methods

### Experimental materials

**Figure 1:**
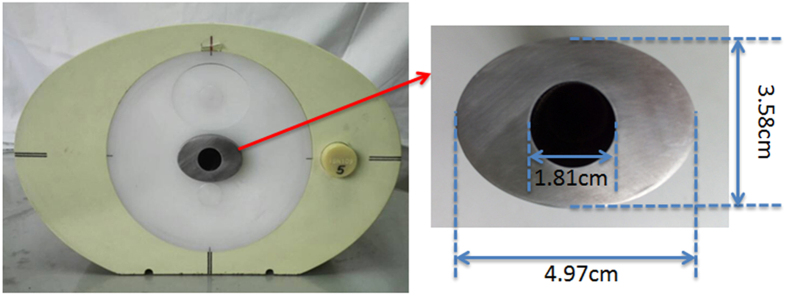
Model.

An intensity-modulated verification phantom (CIRS Company) was used in the experiment (Fig. 1). The center of the phantom was inserted with an elliptical metal rod, creating a cylindrical pinhole in the center. The uncorrected kVCT image of the phantom was obtained through kVCT scanning (SOMATOM Definition Flash CT, Germany Siemens Company) at a scanning voltage of 120 kV. Effective current was automatically generated by CT in axial scanning mode. The width of the collimator was 64 mm × 0.6 mm, and the rotation time of the X-ray tube was 0.5 s/circle. The scanning layer thickness was 2 mm. The MVCBCT image of the phantom was obtained through scanning with MVCBCT (Siemens Artiste accelerator, Germany Siemens Company), with an image acquisition dose of 8 MU. The accelerator rack rotated along the clockwise direction from 180° (total scanning for 360°). The image reconstruction matrix was 512 × 512, the pixel spacing was 0.59 mm × 0.59 mm, and the layer thickness was 0.59 mm. The general CT image presented a depth of 12 bit, ranging from −1024 HU to 3071 HU, but the actual CT value of the metal was higher than 3071 HU. Accurate CT value of the metal can be obtained using the extended 16-bit depth reconstruction image^21^. Metal artifacts are related to the inserted object shape^22^; as such, artifacts caused by a single regular cylindrical metal are small and those caused by an oval metal are large with increasing eccentricity. The experiment used a stainless steel elliptical metal rod, with a density of 7.8 g/cm^3^, elliptical long axis of 4.97 cm, short axis of 3.58 cm, and hole diameter of 1.81 cm in the middle of the cylinder.

### MVCBCT conversion and registration

The linear attenuation coefficient of the same material varies in MVCBCT and kVCT images; as such, the CT values of the two images cannot be directly fused and the MVCBCT image should be transformed into a comparable pseudo-kVCT image. In this paper, a standard “phantom” (30 cm diameter) was reconstructed for MVCBCT and kVCT scanning by using a previously described method^18^. The CT image of the corresponding phantom was obtained, and several tissue-like cylindrical long rods with different densities were inserted in the phantom. The corresponding CT values of the same long rod in the two CT images were recorded. The CT value of the transformational relationship between MVCBCT and kVCT was obtained through linear interpolation.

The scanning fields and spatial positions differed between MVCBCT and kVCT; as such, MVCBCT must be rectified. The MVCBCT image was scaled in MATLAB by using the imresize function with bilinear interpolation to match that the size of the kVCT image. The MVCBCT image was then translated and rotated to coincide its position with that of the kVCT image.

### Fusion of kVCT and MVCBCT

In the fusion of MVCBCT and kVCT, the metal portion in the MVCBCT image was segmented by thresholding (3000 HU) as the metal portion of the fusion image.

**Figure 2:**
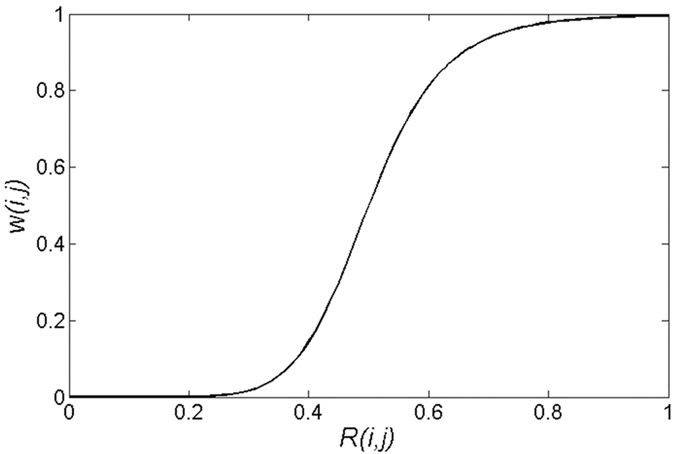
Weighting function corresponding to Eq. (3).

**Figure 3:**
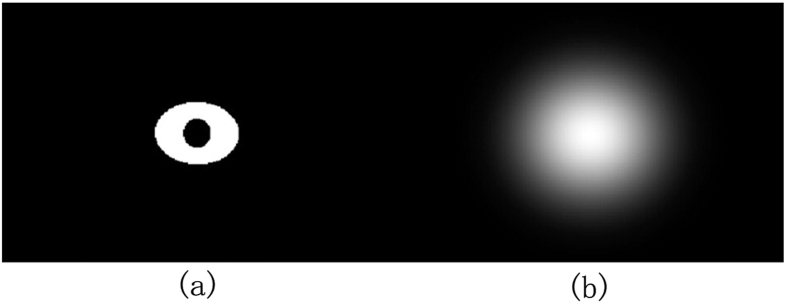
(**a**) metal region, (**b**) weight coefficients *w*_*d*_ (*i, j*).

MVCBCT is minimally affected by the metal artifact and can correctly reflect the CT value in the region surrounding the metal implants. However, MVCBCT exhibits high noise and poor contrast of soft tissues. The MVCBCT value is also less accurate than the value of the kVCT image of normal tissues without interference. Meanwhile, kVCT exhibits high resolution on soft tissues and yields accurate CT value, thereby providing improved contrast. Artifacts caused by metal implants generate severe deviation between the kVCT value and the ground truth in regions with severe artifacts. Based on fusion method proposed by Wang^23^, the CT values of all pixels in the MVCBCT and kVCT images in the present study were fused by a certain weight, as shown in Formula (1).


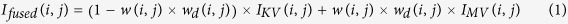


where *I*_*fused*_ is the fused image, *I*_*KV*_ is the kVCT image, *I*_*MV*_ is the MVCBCT image, *w*(*i, j*) and *w*_*d*_(*i, j*) are the weight coefficients of the pixels, and *w*(*i, j*) is obtained using Formulas (2) and (3).


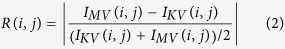



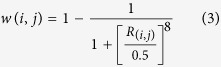


where *R*(*i*, *j*) is the relative deviation of the corresponding pixels in the kVCT and MVCBCT images. If (*I*_*KV*_(*i, j*) + *I*_*MV*_(*I*, *j*)) is zero, the value is set as 1 to justify *R*(*I*, *j*). The relationship between *R*(*I*, *j*) and *w*(*I*, *j*) is shown in Fig. 2. When the relative bias *R*(*I*, *j*) increases, the weight *w*(*I*, *j*) increases. The proportion of the MVCBCT image increases in the fused image. When *R*(*I*, *j*) decreases, the fused image is closer to kVCT. If *R*(*I*, *j*) is high, the MVCBCT and kVCT images considerably differ and the region is likely to be severely affected. MVCBCT is less affected than kVCT and can accurately reflect the real value. Thus, the proportion of MVCBCT should be increased in the fused image. When *R*(*I*, *j*) is low, kVCT is closer to MVCBCT and the influence of artifacts is less. kVCT presents low noise and high resolution. The CT value of the kVCT is more accurate than that of MVCBT; thus, the fused image is closer to the former. *w*_*d*_(*i, j*) is the weight coefficients obtained through Gauss low-pass filter for metal regions segmented in the MVCBCT image. As shown in Fig. 3, *w*_*d*_(*i, j*) decreases with increasing distance from the metal. Serious metal artifacts are located close to metal; thus, the proportion of the MVCBCT image is large near metal and becomes smaller for areas away from the metal.

### Projection modification and image reconstruction

After kVCT was fused with MVCBCT, the metal region was obtained by thresholding segmentation (3000 HU) and replaced with tissue and the CT value in the metal region was set to 0. The prior image was obtained and forward projected to replace the metal trace of the uncorrected kVCT image. Direct replacement causes considerable mutation in the boundary of the metal trace, thereby introducing new artifacts. Thus, linear interpolation was used in projection replacement^24^ to ensure smooth transition of the original projection and surrogate data and thus eliminate jumps on the boundary of the metal projection region.

*P* is the image projection set and contains projection values obtained by *n* detection bins under *m* views. *P*_*k*_ is the projection value of *b* detection bins under α views. *k* = (*a* − 1) × *n* + *b*. The alternative projection method is shown in Formulas (4) and (5).


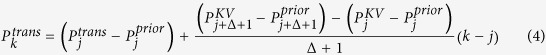



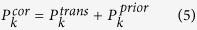


where 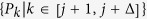 represents the metal trace. *P*_*jj*_ and *P*_j+Δ+1_ are the projection values near the metal trace. *P*^*KV*^ is the uncorrected kVCT projection. *P*^*prior*^ is the projection of the prior image obtained after the fusion. *P*^*cor*^ is the corrected sinogram. FBP reconstruction was performed on the corrected sinogram and the segmented metal portion was added to obtain the corrected CT image.

### Preparation of theoretical CT image

The theoretical CT image was obtained and used as reference to determine the effect of removing metal artifacts. A phantom without a metal bar was scanned through kVCT. Scanning conditions were the same as those mentioned above. kVCT image was obtained without the metal artifact. The metal portion segmented by thresholding (3000 HU) in the MVCBCT image after registration was artificially transplanted into the kVCT image. A fixed CT value of steel was patched in the image in the metal regions. The CT value of the hollow hole at the center of the metal was set as air CT value. The theoretical CT image without the metal artifact was then obtained.

### Comparison with other MAR methods

The proposed method was compared with other MAR methods in terms of the correction effect. Linear interpolation is the most widely used MAR algorithm and is referred to as LIMAR in the present work. To overcome limitations in LIMAR, Meyer^11^ proposed NMAR, in which the sinogram of a prior image is used to normalize the original sinogram before interpolation. The original image is preprocessed by LIAMR, and the prior image is obtained by segmentation of the preprocessing image. This method is referred to as NMAR. Both LIMAR and NMAR are based on the kVCT image only. Paudel^18^ proposed a method that uses MVCT images as prior images in NMAR. In the present work, MVCBCT was used as prior image in NMAR and the method is marked as NMAR-MV. Furthermore, the CT image of a patient with irregular dental fillings was experimented using MAR algorithms to evaluate the correction effects in clinical practice.

### Percentage difference for CT number

The uncorrected kVCT image and corrected image were subtracted from the theoretical CT image to evaluate the metal artifact reduction effect. The percentage difference maps were obtained using Formula (6).


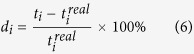


where *t*_*i*_ is the CT number of the uncorrected kVCT image or corrected image, 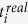 is the CT number of the theoretical CT image mentioned above, and *d*_*i*_ is the percentage difference. If the 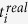 is zero, the value is set as 1 to justify *d*_*i*_.

### Dose distribution in theoretical CT image, uncorrected kVCT image, and corrected image

A radiation treatment plan was formulated in Varian Eclipse (version 11.0) TPS to compare the effect of artifacts on radiation dose distribution. Dose distribution was calculated based on the three CT images: the theoretical CT image without the artifact, the uncorrected kVCT image, and the corrected CT image. Two radiotherapy plans were designed for irradiation. The first plan comprised 0° single irradiation field, with source–skin distance (SSD) = 100 cm. The machine output quantity was 200 MU. The second plan comprised 90° and 270°, which represent two opposed irradiation fields, with source–axis distance (SAD) = 100 cm. The center was set as the center of the metal bar. The machine output quantity of each field was 100 MU, and the irradiation field was 10 cm × 10 cm. The X-ray energy was 6 MV. Dose distribution was calculated using the anisotropic analytical algorithm (AAA).

### Ethical approval

This article does not contain any studies with human participants or animals performed by any of the authors.

## Results and Discussion

### Modified CT images

**Figure 4:**
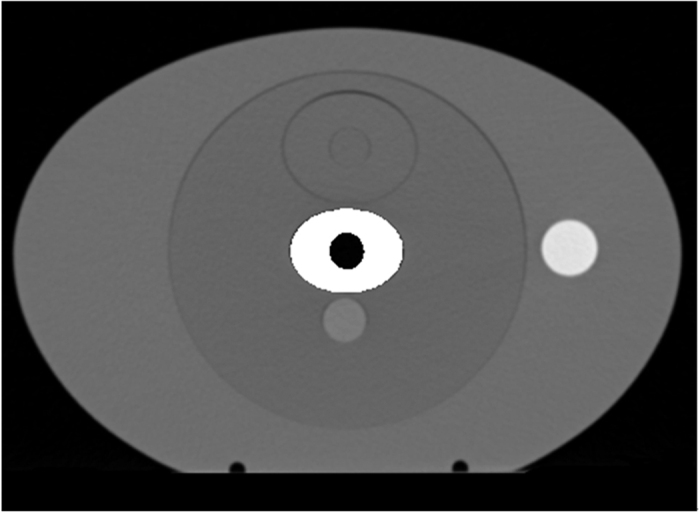
Theoretical CT image.

**Figure 5:**
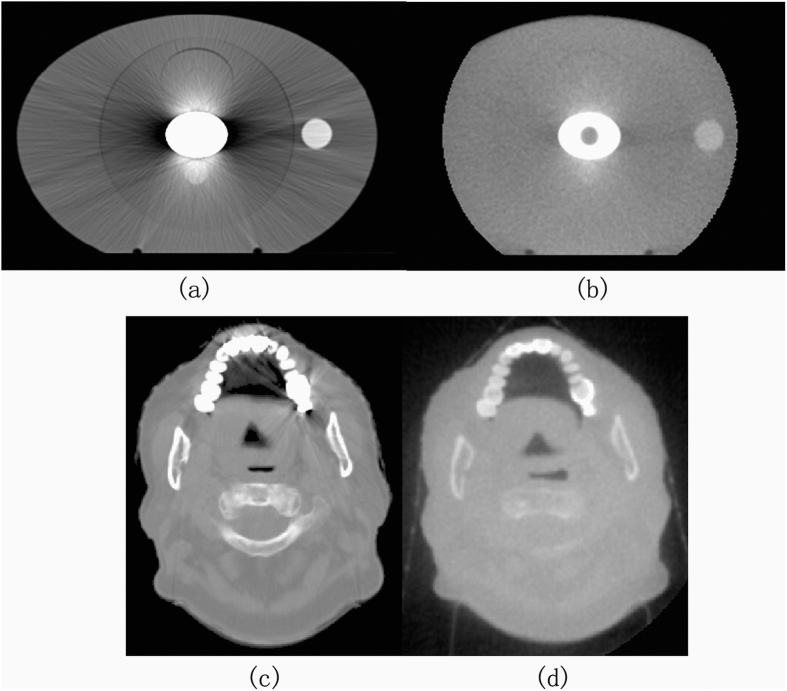
(**a**) Uncorrected kVCT image for phantom, (**b**) MVCBCT image after registration for phantom, (**c**) Uncorrected kVCT image for patient, (**d**) MVCBCT image after registration for phantom.

Figure 4 shows the theoretical CT image without metal artifacts near the metal bar. Figure 5 shows the CT image with the modified metal artifact. Figure 5(a) presents the uncorrected kV image obtained through kVCT scan reconstruction; this image contained severe metal artifacts. Several dark artifacts were also produced on the long elliptical axis near the elliptical metal rod region^22^. The CT values were similar to the air values. Many bright artifacts were produced on the short axis. The air micropore at the center of the metal rod was completely invisible and was misdiagnosed to be the metal portion. Figure 5(b) shows the MVCBCT image after the registration. The metal artifacts were reduced compared with those in the kVCT image. MVCBCT can accurately distinguish the metal structure. An obvious round micropore was found at the center of the metal bar. However, this image featured high noise and inadequate resolution. Figure 5(c) and (d) demonstrate the uncorrected kVCT image and the MVCBCT image of patients with dental fillings. Severe artifacts were observed around the dental fillings in the kVCT image. By contrast, the MVCBCT image displays few artifacts but poor resolution.

**Figure 6:**
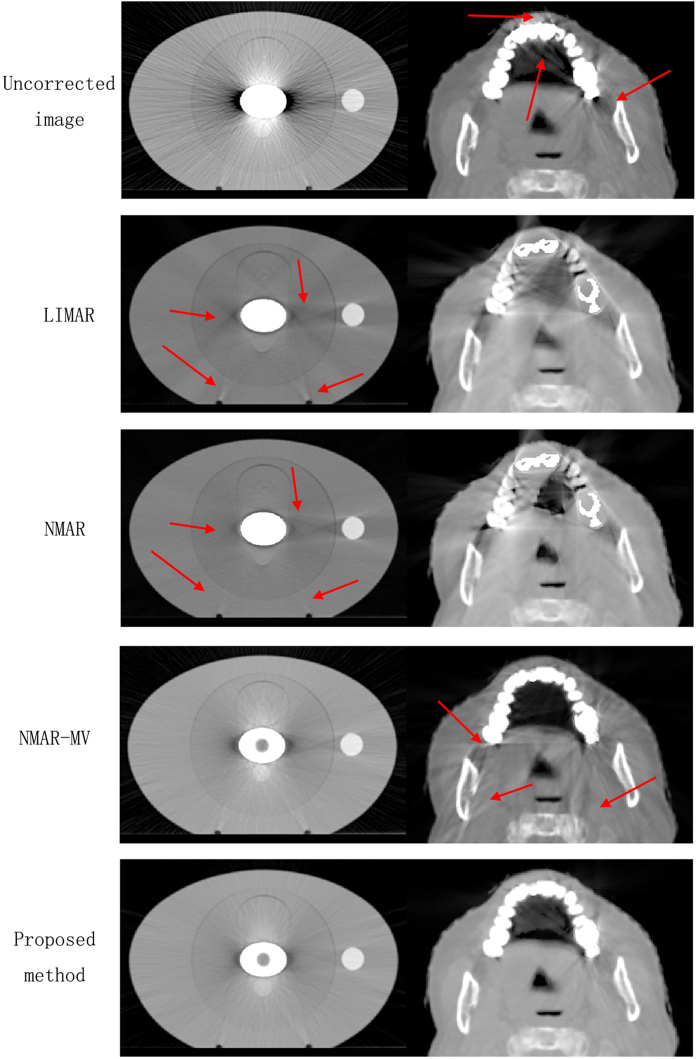
Phantom (left row) with metal implant and patient (right row) with dental fillings.

The results of different MAR methods implemented on phantom and patients are shown in Fig. 6. LIMAR reduced the major artifacts in the phantom except the hole in the metal center. Several artifacts were introduced, which are not severe. The phantom is simple, and CT value distribution is relatively flat. LIMAR is usually applied for homogeneous materials but introduces serious artifacts in the CT images of patients with dental fillings. The head is a highly complicated organ, and its CT values change sharply. LIMAR is thus inapplicable for complicated cases. Similar to LIMAR, NMAR reduces major artifacts, except the hole in metal center in the phantom but generated poor results. In the phantom, NMAR reduces several artifacts, whereas LIMAR introduces new ones (shown as red arrows). LIMAR and NMAR are both based on kVCT images only and cannot recover the hole in metal center because of photon starvation and beam hardening in kVCT. The prior image was obtained by segmentation on the LIMAR image in NMAR. Thus, the correction effect is related to the LIMAR result. NMAR-MV reduced many artifacts and displayed the hole in metal center in the phantom because the MVCBCT image was used as the prior image. For the patient, NMAR-MV reduced artifacts near the dental fillings (shown as red arrows in uncorrected kVCT image), but introduced many artifacts in other locations (shown as red arrows in NMAR-MV). Image registration was implemented on the MVCBCT image according to the kVCT image. However, the MVCBCT image could not precisely match the kVCT image because the structures of patients differ. NMAR-MV directly used the MVCBCT image as prior image for kVCT. The mismatched area between kVCT and MVCBCT resulted in new artifacts in NMAR-MV. Compared with other MAR methods, the proposed algorithm reduced artifacts in both phantom and patient. Minimal artifacts were present near the metal implants, but major artifacts were removed. The quality of the corrected image was evidently improved. In the proposed method, the prior image was obtained by fusing the kVCT and MVCBCT images. The fused area focused on the regions surrounding the metals, where most of the artifacts generated. The fused area can be easily and accurately matched using the metal as reference in registration. The mismatching in other place between kVCT and MVCBCT didnot affect the MAR results in the proposed method.

### Comparison of CT values among the three images

**Figure 7:**
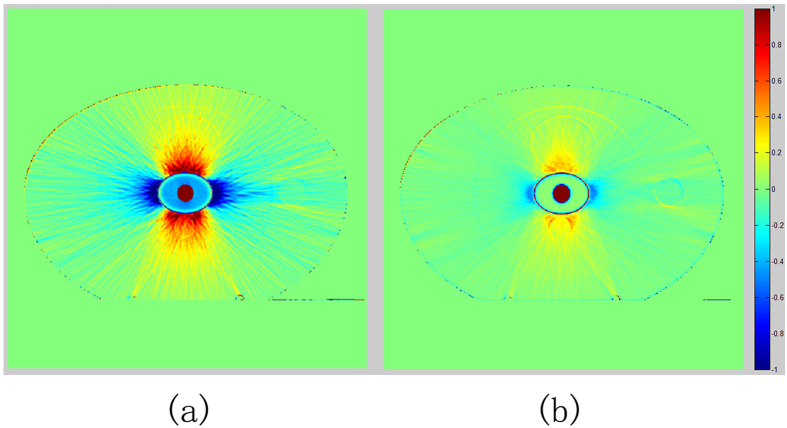
The percentage difference map of CT number. (**a**) represent uncorrected image, (**b**) corrected image.

Artifacts were qualitatively compared between the original CT image and the corrected image. The percentage difference map of CT number is displayed in Fig. 7. Figure 7(a) shows the percentage difference between the original CT image and the theoretical CT image, and Fig. 7(b) presents the percentage difference between the corrected CT image and the theoretical CT image. The difference between the corrected image and the reference is largely reduced compared with that between the original kVCT and the reference.

**Figure 8:**
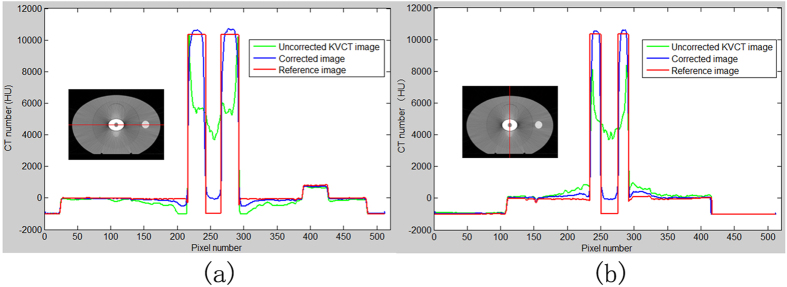
Profiles on the horizontal line (**a**) vertical line (**b**) through the center of the phantom in the three CT images (uncorrected kVCT image, corrected CT image, and reference image).

Figure 8(a) shows the profiles on the horizontal line through the center of the phantom. For the metal portion, the CT value of the uncorrected kVCT image was higher at the metal edge and was close to the real CT value. The CT value at the center of the metal rapidly decreased, and the deviation from the real value was high. The hollow round hole at the center of the metal was not detected in the uncorrected kVCT image. The CT value was higher than 3000 HU and mistaken as the metal portion. The metal portion of the corrected image was segmented from the MVCBCT image, and the CT value was close to the real value. The hollow hole at the center of the metal was evident, and the CT value of the hollow hole was close to water value and about 1000 HU higher than that of the real value, namely, the air CT value. For the non-metallic portion, the CT value of the uncorrected kVCT in the long axis of the ellipse metal was lower than that of the real value. Large differences were observed near the metal. The observed artifact was the dark artifacts shown in Fig. 5(a). The CT value of the corrected image near the metal portion was lower than that of the real value, but the difference was evidently smaller than that in the uncorrected kVCT image. The metal artifact minimally affected the high-density cylindrical rod located far from the metal. The CT value was coincident with the real value in the uncorrected kVCT and the corrected image. Figure 8(b) shows the profiles along the vertical line at the center of the phantom. Similar to Fig. 8(a), the CT value within the phantom considerably deviated between uncorrected CT image and theoretical CT image in Fig. 8(b). The CT value for the non-metal regions of the uncorrected kVCT was higher than that of the real CT value, which was the bright artifact region of the short axis in the elliptical metal.

When kVCT was used to scan the large metal implants, the CT value greatly deviated from the real value because of serious photon starvation, beam hardening, and other effects. MVCBCT had higher ray energy, and its detector could receive photons passing through the metal and could accurately reflect the metal CT value. Thus, the metal CT value of the modified image was more accurate than that of the uncorrected kVCT image. The fusion of MVCBCT and kVCT considerably reduced the dark and bright artifacts surrounding the metal, so minimal metal artifacts appeared on the corrected images based on the fused image. In summary, the quality of the corrected image was considerably improved. The corresponding CT value was close to the real CT value, thereby accurately reflecting the CT values of the metal and normal tissues. CT value in the corrected image is closer to the theoretical CT image.

### Dose distribution of radiotherapy plan

**Figure 9:**
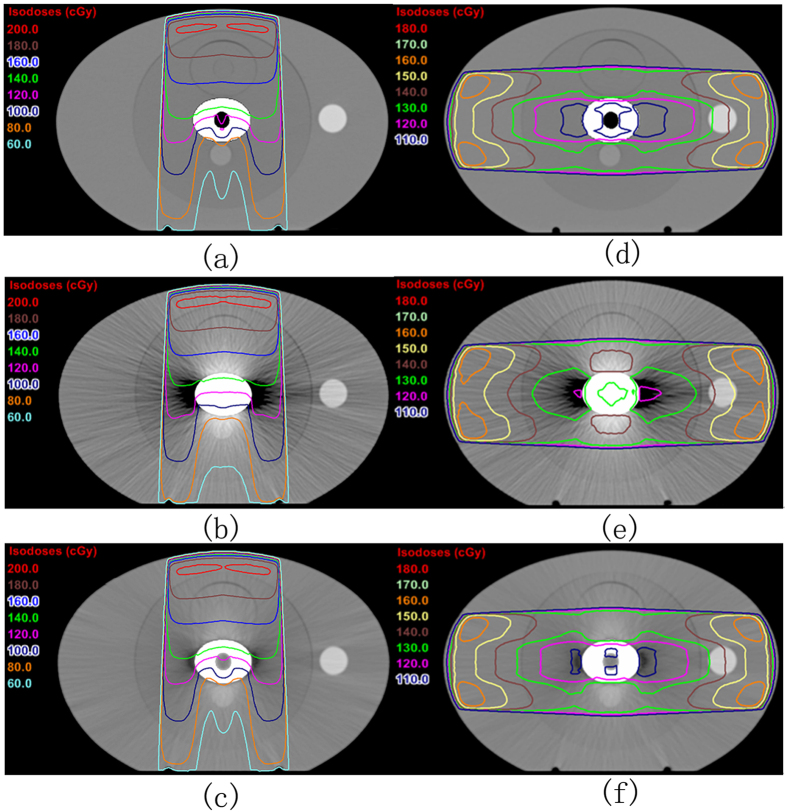
Dose distribution of different CT images in single front-field irradiation plan (**a**), (**b**), (**c**) and horizontal opposed field (**d**), (**e**), (**f**) designed by TPS. (**a**) and (**d**) are theoretical CT images, (**b**) and (**e**) are uncorrected kVCT images, (**c**) and (**f**) are corrected images.

Figure 9 shows the dose distribution calculated from the same radiation therapy plan in the three CT images. Figure 9(a), (b) and (c) represent the dose distributions derived from a single irradiation field. The result showed a large deviation in the isodose curve between the uncorrected kVCT image (Fig. 9(b)) and the theoretical image (Fig. 9(a)). A low concave was found in the 60, 80, and 100 cGy isodose curves, which correspond to the hollow holes of the metal in image (Fig. 9(a)). However, the isodose curve was flat in the kVCT image (Fig. 9(b)). Dose distribution differed in the dark artifact region of both sides of the metal between Fig. 9(a) and (b). The low edges of the 80, 100, and 120 cGy isodose curves in image (Fig. 9(b)) were closer to the edge of the phantom. The dose distribution of the corrected image (Fig. 9(c)) was similar to that of (Fig. 9(a)). Meanwhile, Fig. 9(d) represent the dose distributions of the opposing irradiation fields with the same weight. The result showed an obvious difference in the 120, 130, and 140 cGy isodose curves between the uncorrected kVCT image (Fig. 9(e)) and the theoretical CT image (Fig. 9(d)). The isodose curve in the corrected image (Fig. 9(f)) was close to that of Fig. 9(d). The 110 cGy isodose curve did not exist in Fig. 9(e) but was detected in Fig. 9(d) and (f). In summary, a big difference was observed between the dose distribution calculated based on the uncorrected kVCT image and based on the real value. The corrected image was greatly improved and could accurately reflect the real dose distribution.

**Figure 10:**
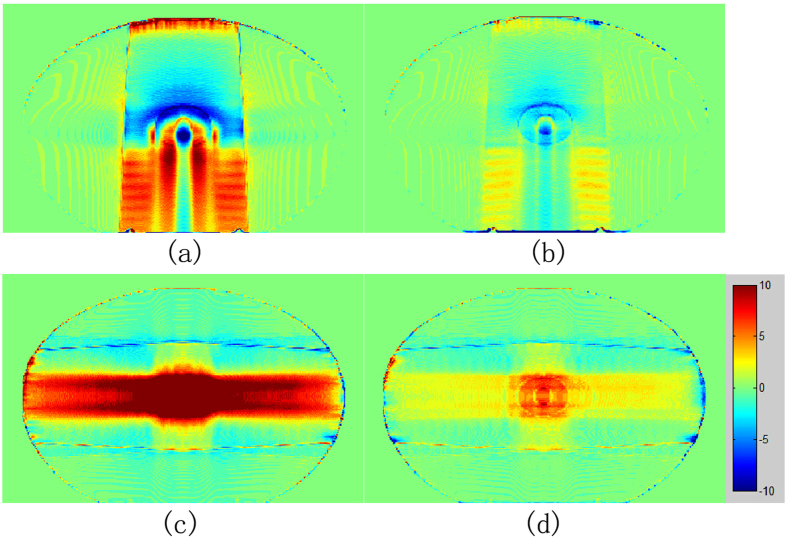
Dose errors for the uncorrected image and corrected image. (**a**) and (**c**) are dose errors for uncorrected image. (**b**) and (**d**) are dose errors for corrected image. The top row is on 0° single irradiation field and bottom row is on 90° and 270° fields. Legends indicate dose in cGy.

**Figure 11:**
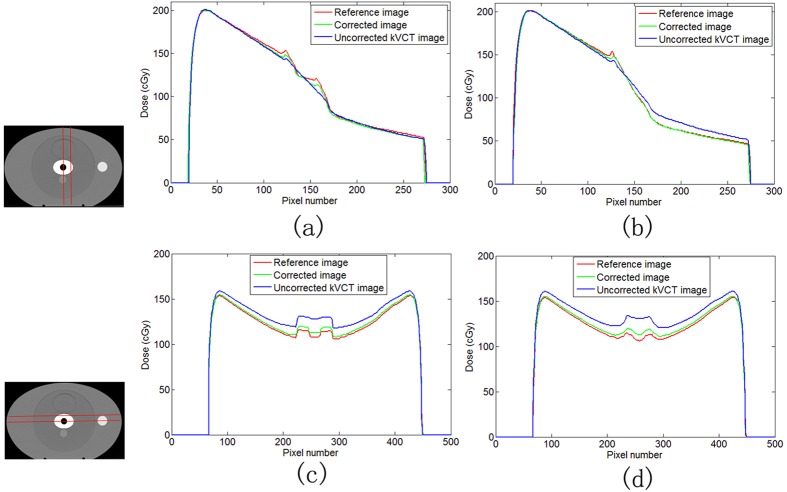
Dose profiles in the phantom. (**a**) and (**b**) are on 0° single irradiation field, (**c**) and (**d**) are on 90° and 270° fields. (**a**) and (**c**) are dose profiles through metal center. (**b**) and (**d**) are dose profiles through metal but not through the hole.

Figure 10 shows the dose distribution errors for the uncorrected and corrected images. Dose errors were computed by subtracting the dose of theoretical CT from corresponding dose of the uncorrected or corrected image. Figure 10(a) and (c) demonstrate large errors in uncorrected image. Dose errors decreased obviously in the corrected image. The dose profiles in the phantom are displayed in Fig. 11. Figure 11(a) and (b) are obtained in 0° single-irradiation field. Figure 11(a) shows the dose profile through the metal center. Two peaks were observed at tissue–metal interface for theoretical CT image and corrected image in Fig. 11(a). The maximum dose error values of 11.8% and 5.1% were obtained for the uncorrected and corrected images, respectively, in Fig. 11(a). The maximum dose error in the corrected image could be due to erroneous CT values of the hole in the center (shown in Fig. 8). Figure 11(b) demonstrates the dose profile through metal (not across the hole). The dose profile of the corrected image precisely coincided with the theoretical CT image and uncorrected image displaying large difference in Fig. 11(b). The maximum dose error values of 17.2% and 3.3% were observed for the uncorrected and corrected image, respectively, in Fig. 11(b). The mean dose errors for the region downstream of metal were 11.8% and 1.0% for the uncorrected and corrected image, respectively, in Fig. 11(b). Figure 11(c) and (d) present the dose profiles through the metalin 90° and 270° irradiation fields. Profile in Fig. 11(c) passes the hole in center, in contrast to that in Fig. 11(d). The dose profile of the corrected image was close to the theoretical image and had similar wave shape in the curves. The dose profile of the uncorrected image differed largely from theoretical image. The mean dose errors were 8.2% and 1.9% for uncorrected and corrected image, respectively, in Fig. 11(c). In Fig. 11(d), the mean dose errors were 9.4% and 2.3% for the uncorrected and corrected image, respectively. Compared with uncorrected image, the corrected image contained reduced dose errors.

The presence of metal artifacts leads to inaccurate CT value in CT images. Inaccurate electron density (or tissue density) was obtained based on the CT value–density conversion curve in TPS, thereby affecting TPS inhomogeneity and providing inaccurate dose distribution, resulting in inaccurate assessment of the absorbed dose of tumor and normal tissues. Therefore, reduction of metal artifacts is a key factor to enhance the accuracy of radiotherapy calculation in TPS for patients with metal implants. Reduction of metal artifacts provides accurate dose distribution for medical personnel and improves the accuracy of evaluating radiotherapy effects and side effects on normal tissues. Metal artifact removal also improves the image quality and decreases the misdiagnosis of medical personnel.

## Conclusion

In this paper, a novel method of metal artifact correction is proposed based on traditional method of using prior images. The fusion of the MVCBCT and the uncorrected kVCT images was obtained as the prior image. The proposed method combines the advantages of the MVCBCT image, including less metal artifacts and accurate CT value of the metal, with the advantages of the kVCT image, including high soft tissue contrast resolution and low noise. Compared with that of the uncorrected kVCT image, the quality of the corrected image was greatly improved, the metal artifact was obviously reduced, and the CT value was more accurate. In the corrected image, normal tissue structures were preserved and artifacts were removed. The dose distribution in the radiotherapy plan showed that metal artifacts considerably affected dose distribution, which differed largely between the uncorrected kVCT and the theoretical CT images. Image dose was greatly improved after correction and approximated the real dose distribution. The proposed method for metal artifact correction improved the quality of the CT image and the accuracy of dose distribution in radiotherapy.

## Additional Information

**How to cite this article**: Liugang, G. *et al*. Metal artifact reduction through MVCBCT and kVCT in radiotherapy. *Sci. Rep.* **6**, 37608; doi: 10.1038/srep37608 (2016).

**Publisher’s note**: Springer Nature remains neutral with regard to jurisdictional claims in published maps and institutional affiliations.
